# A Cheonjiin Layout Mental Speller: Developing a Simple and Cost-Effective EEG-Based Brain–Computer Interface System

**DOI:** 10.3390/s26072265

**Published:** 2026-04-07

**Authors:** Ji Won Ahn, Gi Yeon Yu, Seong-Wan Kim, Young-Seek Seok, Kyung-Min Byun, Seung Ho Choi

**Affiliations:** 1 Department of Biomedical Engineering, Yonsei University, Wonju 26493, Republic of Korea; jw010601@yonsei.ac.kr (J.W.A.); dbrldus074@yonsei.ac.kr (G.Y.Y.); 2 Department of Agricultural Biology, National Institute of Agricultural Sciences, Rural Development Administration, Wanju 55365, Republic of Korea; tarupa@korea.kr; 3 Gangwon-do Agricultural Product Registered Seed Station, Chuncheon 24410, Republic of Korea; air5738@korea.kr; 4 Department of Biomedical Engineering, College of Electronics and Information, Kyung Hee University, Yongin 17104, Republic of Korea; 5 Department of Electronics and Information Convergence Engineering, Kyung Hee University, Yongin 17104, Republic of Korea; 6 Department of Integrative Medicine, Major in Digital Healthcare, Yonsei University College of Medicine, Seoul 06229, Republic of Korea

**Keywords:** brain–computer interface (BCI), electroencephalography (EEG), speller system, Cheonjiin keyboard, directional input

## Abstract

A brain–computer interface (BCI) enables direct communication between the brain and external devices by translating neural activity into executable control commands. Among electroencephalography (EEG)-based paradigms, steady-state visual evoked potential (SSVEP) is widely adopted due to its high signal-to-noise ratio, robustness, and minimal calibration requirements. While SSVEP-based spellers have been extensively investigated, many existing systems rely on high-channel-density EEG recordings and computationally complex processing pipelines, and are primarily designed for alphabetic input structures. In this study, we present an SSVEP-based Korean speller that integrates the Cheonjiin keyboard layout to support intuitive composition of Hangul syllables. The proposed system adopts a simple configuration, employing only five visual stimulation frequencies (6.67–12 Hz) and two occipital EEG channels (O1 and O2), with real-time frequency recognition performed using canonical correlation analysis (CCA) within a 1.5 s sliding window. EEG signals were acquired at 200 Hz using an OpenBCI Ganglion board, band-pass filtered (5–45 Hz), and processed with harmonic sinusoidal reference templates for multi-frequency classification. The proposed interface generates five control commands (up, down, left, right, and select), enabling directional cursor navigation and character confirmation on a 4 × 4 virtual Cheonjiin keyboard. Experimental validation with three healthy participants demonstrated an average classification accuracy of approximately 82% and an information transfer rate (ITR) of 31.2 bits/min. Frequency-domain analysis revealed clear spectral peaks at the stimulation frequencies and their harmonics, indicating reliable SSVEP responses. The proposed system employs a simple two-channel configuration integrated with a Korean language-specific input structure, demonstrating that reliable SSVEP-based communication can be realized without computationally intensive algorithms or high-cost EEG acquisition systems. These findings demonstrate that reliable SSVEP-based communication can be achieved using a low-channel configuration without reliance on high-cost EEG equipment.

## 1. Introduction

A brain–computer interface (BCI) serves as a communication bridge between the human brain and external devices by translating neural activity into control commands [1,2,3]. Over the past few decades, BCI research has expanded rapidly with the aim of improving quality of life, particularly for individuals with severe motor impairments. For example, individuals with locked-in syndrome are fully conscious but experience near-complete paralysis, retaining only limited voluntary eye movements [4,5]. In such cases, unlike conventional assistive technologies that depend on residual motor function, BCI bypasses the peripheral neuromuscular system and directly decodes brain signals, providing a motor-independent pathway for communication. Accordingly, interest in BCI research has steadily increased, leading to rapid technological and methodological advancements. These developments have broadened the functional scope of BCI systems beyond clinical settings to diverse real-world applications.

Electroencephalography (EEG) is one of the most commonly used signal acquisition modalities in BCI systems due to its non-invasive nature and high temporal resolution [6,7]. Such EEG-based control frameworks have enabled a wide range of practical applications, including robotic arms, wheelchairs, computer cursors, web browsers, and speller systems for text entry. The realization of these applications depends on the selection of specific neurophysiological signal paradigms. Among EEG-based paradigms, visual evoked potentials (VEPs) reflect electrical responses elicited in the visual cortex following visual stimulation and have been widely utilized due to their robust, stimulus-locked characteristics [8,9].

The steady-state visual evoked potential (SSVEP) is a continuous oscillatory response elicited when a user focuses attention on a repetitive visual stimulus flickering at a constant frequency. Accordingly, an SSVEP-based BCI is widely regarded as advantageous for practical applications, as it typically exhibits a high signal-to-noise ratio (SNR), requires minimal user training and calibration, and enables a relatively high information transfer rate (ITR) [10].

SSVEP-based speller systems have been extensively investigated since the early 2000s. In 2002, Cheng et al. introduced an SSVEP-based BCI speller, which laid the foundation for subsequent research in this area [11]. Since then, numerous studies have sought to improve classification accuracy, information transfer rate, and system usability [12,13]. In this process, a wide range of methodological and technological refinements have been introduced, reflecting continuous efforts to optimize stimulation design, signal acquisition strategies, and decoding approaches. Most existing SSVEP-based speller systems have relied on multi-channel EEG setups, incorporating signals from multiple occipital and parietal electrodes for frequency decoding [14,15,16]. Other studies have employed higher stimulation frequencies and reduced inter-frequency spacing to increase the number of distinguishable targets within a constrained frequency band [14,17,18]. In addition, signal processing approaches have evolved from conventional spectral analysis toward more advanced decoding frameworks designed to improve classification reliability under real-time conditions. In particular, canonical correlation analysis (CCA) has been widely adopted for frequency recognition due to its calibration-free properties and computational efficiency, and subsequent extensions such as filter bank CCA (FBCCA) have utilized sub-band decomposition and harmonic information to further enhance classification performance [19,20].

In recent years, SSVEP-based BCI research has increasingly incorporated advanced computational approaches. Deep learning-based EEG decoding methods, including convolutional neural networks (CNNs) and recurrent neural networks (RNNs), have been explored to improve classification performance and robustness under complex conditions [21,22,23]. In addition, hybrid BCI systems integrating multiple physiological signals, such as electrooculography (EOG) or electromyography (EMG), have been proposed to enhance control reliability and expand the number of available commands [24,25,26,27]. Furthermore, modern speller paradigms, including asynchronous interfaces and adaptive stimulation strategies, have been introduced to improve usability and reduce user fatigue in real-world environments [28,29].

Despite these advances, several limitations remain in current SSVEP-based speller systems. From a technical perspective, many SSVEP-based spellers rely on multi-channel EEG configurations to achieve robust frequency recognition, thereby requiring elaborate signal acquisition and processing procedures [14,15,16,30,31]. Also, substantial inter-subject and session-to-session variability have been reported, often necessitating subject-specific calibration procedures [32,33,34]. Although advances in stimulation design and signal processing have improved classification accuracy and information transfer rates, practical challenges related to calibration procedures, hardware setup, and overall usability continue to hinder real-world deployment [35]. Furthermore, the reliance on continuous visual stimulation and sustained visual fixation may induce visual fatigue and increase cognitive load, particularly during prolonged operation, thereby limiting long-term usability [36].

From a linguistic perspective, the majority of BCI speller systems have been developed primarily for alphabetic languages such as English [37]. In contrast, non-alphabetic or syllabic writing systems have received comparatively limited attention in BCI speller research. Consequently, keyboard layouts that explicitly reflect the structural and compositional characteristics of these languages have not been systematically incorporated into existing speller designs. Previous BCI speller studies have explored text entry for non-alphabetic languages, such as Chinese and Japanese, typically using large character sets or hierarchical selection strategies. While these approaches enable language-specific input, they often rely on a large number of selectable targets or multi-step selection processes, which can increase system complexity and user burden under limited command conditions [38,39]. Unlike English, which follows a linear alphabetic structure, Hangul—the writing system of the Korean language—has a compositional syllabic structure in which consonants and vowels are combined to form syllable blocks [40,41]. Under a direction-based control paradigm with a limited number of selectable targets, keyboard layout efficiency becomes a critical determinant of overall input performance.

To enable efficient Korean text entry under a limited command set, it is necessary to consider keyboard layouts that reflect the structural characteristics of the Hangul writing system. Unlike alphabetic languages, Hangul is composed of syllabic blocks formed by the sequential combination of an initial consonant, a medial vowel, and, optionally, a final consonant. This compositional structure requires multiple input steps for a single character, making conventional keyboard layouts less suitable for interfaces with restricted input commands. However, applications to Korean text entry remain limited, particularly in combination with structured keyboard layouts such as Cheonjiin [42,43].

Among Korean text input systems, the most widely used keyboard layouts include QWERTY-based keyboards and the Cheonjiin layout. The Cheonjiin layout, introduced by LG Electronics in 2000, is based on the conceptual elements of heaven (ㆍ), earth (ㅡ), and human (ㅣ), enabling character composition through combinations and sequential inputs using a reduced set of keys [44]. Under the same limited command environment, QWERTY-based input requires a relatively large number of cursor movements to navigate between distributed keys and select individual characters, whereas the Cheonjiin layout allows for character generation through a smaller set of targets with sequential selections. For example, entering the Korean word “안녕하세요” (meaning “hello”) requires 48 cursor movements in a QWERTY layout but 34 movements in the Cheonjiin layout, highlighting its suitability for systems with a limited number of control commands.

To address these limitations, our study proposes a simple and cost-effective SSVEP-based speller framework that integrates the Cheonjiin keyboard layout. The system is designed for practical deployment under minimal hardware and computational constraints within a direction-based control paradigm. Specifically, rather than relying on multi-channel EEG configurations, the system employs only two occipital electrodes (O1 and O2) to capture SSVEP responses from the visual cortex, thereby reducing setup complexity while preserving reliable frequency detection. Five stimulation frequencies (6.67, 7.5, 8.57, 10, and 12 Hz) were employed and mapped to directional cursor commands (up, down, left, and right) as well as a selection command. Frequency recognition was performed using a conventional canonical correlation analysis (CCA), without incorporating additional computationally intensive extensions. This design enables implementation using low-cost EEG hardware and minimizes elaborate signal acquisition and calibration procedures.

Our study presents the architectural design and implementation of the proposed SSVEP-based speller and presents a quantitative evaluation of its performance in two experimental settings. The first experiment assesses the accuracy of directional command recognition, whereas the second examines practical Korean text entry performance under real input conditions. System performance was evaluated using standard classification metrics, including the confusion matrix, accuracy, precision, recall, and F1-score. The developed speller provides an alternative communication pathway for individuals with locked-in syndrome, particularly those who rely on Korean text for daily communication. Furthermore, its simple system architecture and low-resource implementation facilitate practical deployment and enable integration into broader assistive technology platforms, such as rehabilitation systems and intelligent interfaces.

## 2. Materials and Methods

### 2.1. Participants

Three healthy participants (aged 24 ± 2 years) with normal or corrected-to-normal vision volunteered for both the offline and online SSVEP experiments. None of the participants had a history of neurological disorders or photosensitive epilepsy, and they reported either no prior experience or only minimal familiarity with SSVEP-based BCI systems. Participants were required to have the ability to communicate in Korean and were familiar with the Cheonjiin keyboard layout, a common text input method on Korean mobile devices. The exclusion criteria covered any factors that could potentially compromise the integrity of the experiment.

Before the experiment, individuals received a detailed explanation of the experiment procedures and subsequently reviewed and signed an informed consent form, which included notification of their right to withdraw at any time without consequence. Personal information was anonymized to ensure participant confidentiality. The study was approved by the Ethics Committee of the Yonsei University Wonju Institutional Review Board (1041849-202511-BM-254-03).

### 2.2. Experimental Design

#### 2.2.1. Experimental Setup

The experiment took place in a quiet indoor setting devoid of electronic disturbance, with light and other environmental factors kept consistent to ensure the quality of signals and sustain participant focus. Participants sat in a comfortable chair with their upper body in a relaxed position, and the status of the electroencephalography (EEG) electrodes was checked both before and after the experiment to guarantee consistent recording.

**Figure 1:**
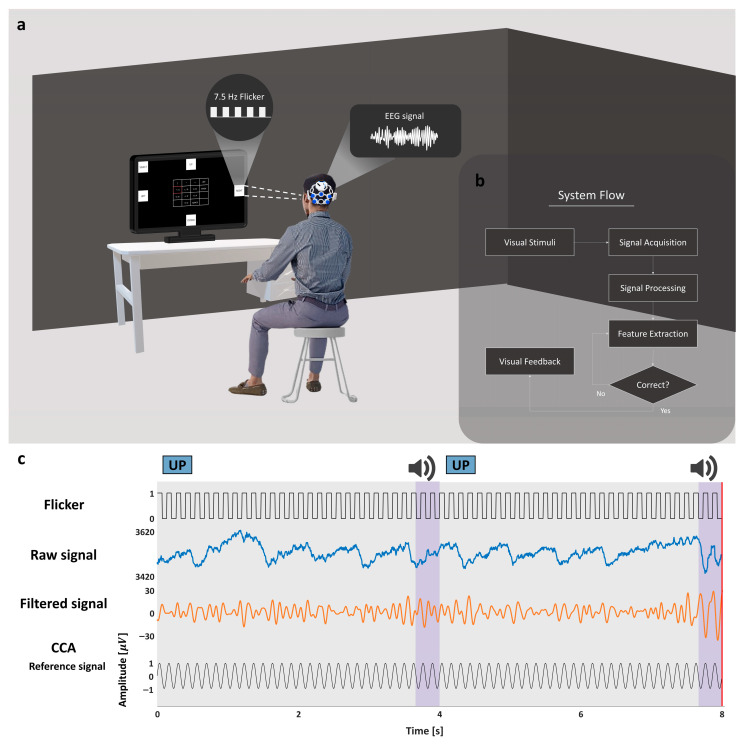
The experimental paradigm and system framework of the proposed SSVEP-based Cheonjiin speller. (**a**) An overview of the experimental configuration; (**b**) a system flow diagram illustrating signal acquisition, preprocessing, real-time SSVEP decoding, and command generation; (**c**) an illustration of visual stimulation and corresponding EEG responses, including frequency-coded visual stimulation, raw EEG recordings, filtered signals, and corresponding CCA reference signals used for correlation-based frequency recognition.

The overall experimental configuration is illustrated in Figure 1, providing an overview of the experimental setup and signal acquisition framework. EEG signals were recorded using a two-channel EEG system, and the system architecture enabled real-time decoding of SSVEP responses during task performance. The Cheonjiin keyboard was displayed on the monitor, displaying cursor movements and character input feedback in real time for immediate response. Participants were instructed to control the system by staring at the flickers throughout the experiment. All selections were generated voluntarily without any external prompting, and participants autonomously determined when to initiate each command.

#### 2.2.2. System Design

An EEG-based Cheonjiin speller was developed using two occipital electrodes (O1 and O2), with POz serving as the reference. Based on the acquired EEG signals, the system produced five control commands consisting of four directional inputs designated as UP, DOWN, LEFT and RIGHT, along with a selection command referred to as SELECT. EEG signals were recorded using an OpenBCI Ganglion board integrated into the overall system architecture. The digitized signals were transmitted to a computer where real-time preprocessing, feature extraction, and command classification were executed. Processed outputs were presented on a monitor through a graphical user interface that delivered continuous visual confirmation of user commands.

The entire system was implemented on a computer running Windows, equipped with a 12th Gen Intel Core i7-1260P processor and 16 GB RAM. Signal acquisition and processing were implemented in Python (v3.10.8) using the BrainFlow library (v5.19.0) in conjunction with NumPy (v2.2.6) and scikit-learn (v1.7.2).

#### 2.2.3. Monitor Design

**Figure 2:**
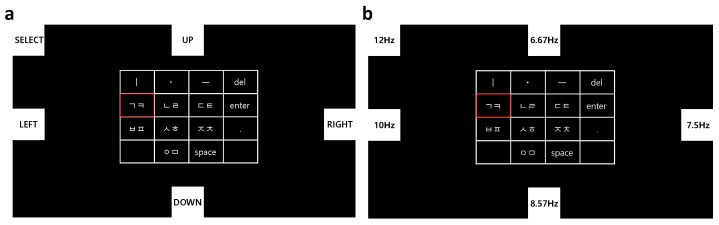
The graphical user interface and keyboard configuration of the proposed EEG-based Cheonjiin speller. (**a**) Screen display of the EEG-based Cheonjiin system, where the red box indicates the current cursor position; (**b**) Cheonjiin keyboard layout with each stimulus labeled by its corresponding frequency (Hz).

The EEG-based Cheonjiin speller system was developed in a Python environment using the PyQtGraph and Pygame libraries. Figure 2 illustrates the graphical user interface of the proposed EEG-based Cheonjiin speller, integrating frequency-coded flicker stimuli with the Cheonjiin keyboard layout. Five distinct flicker frequencies were assigned to the control commands to evoke SSVEP responses: 6.67 Hz for UP, 7.5 Hz for RIGHT, 8.57 Hz for DOWN, 10 Hz for LEFT, and 12 Hz for SELECT. The stimulation frequencies were selected based on integer divisions of a 60 Hz refresh rate to ensure stable flicker generation, maintaining consistency with the display refresh cycle. The keyboard layout follows a grid structure with four rows and four columns, providing a total of fourteen input keys arranged at the center of the interface. The top row contains the vowel components ㅣ, ㆍ, and ㅡ along with a delete key. The remaining rows contain consonant keys and additional function keys including space, enter, and a period. The key selected by the user is marked with a red outline to indicate its active state.

Visual stimuli were presented on a 27-inch LCD monitor (SE2725HG, Dell Inc., Round Rock, TX, USA) with a resolution of 1920 × 1080 pixels. The monitor supports a maximum refresh rate of 200 Hz; however, it was configured to operate at 60 Hz for the experiments. To provide immediate visual and auditory feedback upon command detection, the system briefly changed the color of the corresponding tile and simultaneously generated a short auditory cue whenever a control command was identified. This feedback allowed participants to confirm correct command recognition before proceeding to the next action.

### 2.3. Signal Acquisition and Preprocessing

#### 2.3.1. Signal Acquisition

In this study, EEG signals were recorded from the occipital region using O1 and O2 as the primary recording sites. According to the international 10–10 electrode placement system, O1 and O2 represent standardized occipital scalp locations associated with visual cortical activity and were selected for their functional relevance to visual processing. These electrodes are commonly employed in SSVEP studies and are known to yield reliable and reproducible occipital responses. Moreover, the high reproducibility of occipital responses under repeated visual stimulation makes them suitable for sustained operation in EEG-based interface applications.

Two occipital electrodes were selected to capture SSVEP responses from bilateral visual cortical regions. This configuration allows for more stable correlation estimation by accounting for inter-channel variability in signal amplitude and phase, and reduces the impact of localized noise or electrode-specific signal degradation compared to a single-channel setup. Therefore, a two-channel configuration was adopted to ensure stable SSVEP detection while maintaining a relatively low-complexity system design.

EEG signals were recorded using passive dry comb EEG electrodes (Dry EEG Comb Electrodes, OpenBCI Inc., Brooklyn, NY, USA). To enhance electrical conductivity and reduce electrode–scalp impedance, saline-soaked sponge inserts were applied to the electrode tips during recording, resulting in a hybrid dry–wet electrode configuration. The electrodes were connected to the EEG signal acquisition device (Ganglion board, OpenBCI Inc., Brooklyn, NY, USA), which incorporates an analog front end (MCP3912) with built-in amplification and 24-bit A/D conversion [45].

**Figure 3:**
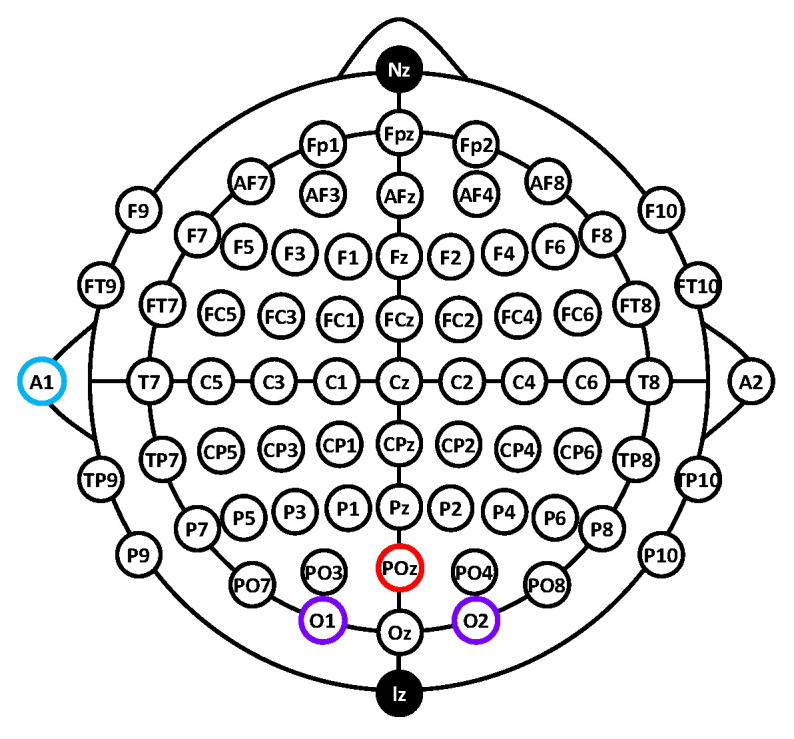
EEG electrode placement. Purple circles denote the EEG recording sites (O1 and O2), the red circle denotes the reference electrode (POz), and the blue circle denotes the ground electrode (A1, left earlobe).

EEG signals were acquired using a multi-channel EEG recording system with continuous data acquisition throughout the experimental session. As illustrated in Figure 3, two-channel EEG signals were recorded from O1 and O2 and referenced to POz, with the ground electrode placed at A1 (left earlobe). Electrode impedances were monitored prior to and during the experiment and were maintained below 50 kΩ, typically around 20 kΩ, to ensure stable recording conditions. The EEG data were sampled at 200 Hz and continuously streamed to a computer for real-time processing.

#### 2.3.2. Signal Preprocessing

The analog EEG signals acquired from the two channels were amplified and digitized at a sampling rate of 200 Hz using an OpenBCI Ganglion board, and the digitized data were transmitted to a Python environment via the BrainFlow library. To ensure stable and reliable system operation, the acquired signals were subjected to a sequence of preprocessing and feature extraction steps before being used for cursor control.

EEG signal preprocessing and online decoding were implemented in Python to ensure real-time compatibility with the OpenBCI Ganglion board. Two-channel EEG signals recorded from the occipital region (O1 and O2) were continuously streamed using the BrainFlow library and processed using standard Infinite Impulse Response (IIR) filtering routines optimized for low-latency operation. For each channel, the raw EEG signals were first detrended to remove DC offsets, followed by a second-order Butterworth band-pass filter (5–45 Hz) to preserve frequency components relevant to steady-state visual evoked potential (SSVEP) while attenuating slow drifts and high-frequency noise. To further suppress power-line interference and hardware-induced artifacts, additional band-stop filters were sequentially applied at 48–52 Hz and 58–62 Hz. Dedicated artifact removal techniques were not applied during online processing.

The entire decoding process was implemented in a continuous real-time pipeline. Filtered EEG signals were segmented using a 1.5 s sliding analysis window with updates every 50 ms for continuous decoding, which inherently introduces a decision latency of approximately 1.5 s. Within each window, canonical correlation analysis (CCA) was employed to quantify the similarity between the preprocessed EEG signals and a set of predefined reference templates. The reference signals consisted of orthogonal sine–cosine pairs generated at the stimulation frequencies (6.67, 7.5, 8.57, 10, and 12 Hz), enabling phase-invariant detection of frequency-specific SSVEP responses.

This processing pipeline was designed to operate with minimal computational overhead under the resource constraints of the Ganglion board, achieving real-time performance without reliance on computationally intensive machine learning models. The signal processing pipeline consists of windowed CCA, channel-wise maximum correlation selection, and temporal accumulation, which provide basic handling of noise, inter-channel variability, and momentary signal fluctuations.

#### 2.3.3. Feature Extraction

Frequency-domain correlation features were selected for their proven robustness in SSVEP-based EEG classification and suitability for real-time implementation. Since the early development of SSVEP-based speller systems in the 2000s, correlation-based decoding strategies have been widely adopted as a reliable framework for frequency identification [11,46]. Among various candidates, canonical correlation coefficients derived from canonical correlation analysis (CCA) were chosen based on their strong physiological interpretability and consistent discriminative performance reported in prior SSVEP studies. CCA-based features have been extensively employed in EEG-based interface systems because they directly quantify the similarity between recorded neural responses and stimulus-locked reference signals, enabling reliable frequency identification without explicit training procedures [14,19,47].

Specifically, CCA evaluates the linear relationship between multi-channel EEG signals and sinusoidal reference templates constructed at predefined stimulation frequencies. Because SSVEP responses are characterized by phase-locked oscillatory components at the stimulus frequency, correlation-based metrics provide a direct and physiologically meaningful representation of neural entrainment strength. A higher canonical correlation coefficient indicates stronger synchronization between the recorded EEG activity and a particular visual stimulus [46].

Although alternative approaches such as filter-bank CCA (FBCCA), power spectral density (PSD)-based peak detection, or deep learning-based classifiers (e.g., CNN- or LSTM-based architectures) have been proposed in recent EEG studies, they were not adopted in this work due to their increased computational complexity and limited suitability for low-resource, real-time operation on the OpenBCI Ganglion platform. Previous studies have explored multi-band processing and deep learning-based approaches for SSVEP classification, which involve additional processing steps such as multi-band signal decomposition or model training. Filter bank-based methods also require decomposition into multiple frequency bands and repeated feature extraction across sub-bands, leading to increased computational complexity relative to conventional single-band CCA approaches [13,23,48,49].

Let $X\in R^{N\times C}$ denote the multi-channel EEG signal within a given analysis window, where N is the number of samples and C is the number of channels. For each stimulation frequency $f_{k}$, a corresponding reference signal matrix $Y_{k}\in R^{N\times 2}$ was constructed using sine–cosine pairs:

(1) $$Y_{k}=\left[\begin{array}{cc} sin(2\pi f_{k}t_{1}) & cos(2\pi f_{k}t_{1})\\ sin(2\pi f_{k}t_{2}) & cos(2\pi f_{k}t_{2})\\ \vdots & \vdots \\ sin(2\pi f_{k}t_{N}) & cos(2\pi f_{k}t_{N}) \end{array}\right]$$

CCA seeks projection vectors $w_{x}$ and $w_{y}$ that maximize the correlation between the projected signals:

(2) $$\rho_{k}=\underset{w_{x},w_{y}}{\max}\frac{\langle Xw_{x},Y_{k}w_{y}\rangle }{\left\|Xw_{x}\right\|\;\left\|Y_{k}w_{y}\right\|}$$

where $\rho_{k}$ represents the canonical correlation coefficient associated with frequency $f_{k}$. The absolute value of $\rho_{k}$ was used to quantify frequency-specific similarity. This formulation can be equivalently expressed in matrix form as follows:

(3) $$\rho_{k}=\underset{w_{x},w_{y}}{\max}\frac{w_{x}^{T}X^{T}Y_{k}w_{y}}{\sqrt{w_{x}^{T}X^{T}Xw_{x}\;\cdot \;w_{y}^{T}Y_{k}^{T}Y_{k}w_{y}}}$$

For each stimulation frequency, the canonical correlation coefficient $\rho_{k}$ is computed, and its absolute value is used as a measure of similarity between the recorded EEG signals and the reference signals. The target frequency is then determined by selecting the frequency that yields the maximum correlation:

(4) $$\hat{f}=arg\;\underset{k}{\max}\left|\rho_{k}\right|$$

This approach enables the detection of SSVEP responses by capturing frequency-specific phase-locked neural activity. Correlation coefficients were computed independently for each channel, and the maximum correlation across channels was retained as the representative feature for each stimulus frequency. This channel-wise maximum strategy preserves dominant responses by selecting the strongest correlation across channels for each stimulus frequency.

#### 2.3.4. Classification

The CCA-based correlation features extracted from the EEG signals were used to classify user intent in real time. For each analysis window, canonical correlation coefficients were computed between the EEG signals recorded at O1 and O2 and the reference signals corresponding to the five stimulation frequencies. Correlation values were calculated independently for each channel, and the maximum correlation across channels was selected to form a single correlation vector representing the current window.

For decision-making, correlation values were accumulated over a fixed collection duration of 1.5 s to enhance classification stability and reduce transient fluctuations. Following each confirmed detection, a 1 s ignore interval was introduced to prevent repetitive selections and ensure stable command generation. A stimulus frequency was considered valid only if its correlation coefficient exceeded a predefined threshold (r ≥ 0.25) in at least 30% of consecutive decoding updates within the collection window, where these threshold parameters were empirically determined based on preliminary experiments. Among valid candidates, the frequency with the highest mean accumulated correlation was selected as the final classification result. If no frequency satisfied this consistency criterion, no command was issued. Following each confirmed classification, a short ignore interval was applied to prevent repeated detections of the same stimulus.

Based on the classified stimulus frequency, user actions were mapped to five discrete control commands. The frequencies of 6.67, 7.5, 8.57, and 10 Hz were mapped to the UP, RIGHT, DOWN, and LEFT commands, respectively, while 12 Hz was mapped to the SELECT command. Once a frequency was classified, the corresponding command was immediately executed to update the cursor position or confirm a selection within the Cheonjiin keyboard interface.

### 2.4. Experimental Procedure

Prior to the main experiments, a preliminary study was conducted to determine the optimal analysis window size for SSVEP decoding. This preliminary experiment was performed using the same participants who later took part in the main experiments. The analysis window was varied from 0.5 s to 3.0 s in increments of 0.5 s. For each window condition, five directional commands (UP, DOWN, LEFT, RIGHT, and SELECT) were executed twice, and classification performance was evaluated using accuracy and information transfer rate (ITR). Based on this procedure, a 1.5 s analysis window was selected for the main experiments to balance recognition reliability and response latency.

Two experiments were conducted to assess the feasibility and usability of the proposed EEG-based Cheonjiin speller system. The first experiment, referred to as the single-step command recognition evaluation, investigated the system’s ability to accurately recognize and classify SSVEP-based directional commands. The second experiment, the multi-step Korean text composition evaluation, examined the practical usability of the system during continuous Hangul text entry tasks.

The single-step command recognition (Experiment 1) was designed to quantify the recognition accuracy of individual directional commands under controlled conditions, whereas the multi-step Korean text composition (Experiment 2) focused on assessing system usability and performance during sustained operation in a realistic text entry scenario. Together, these experiments provided a complementary evaluation of overall system performance by relating command recognition accuracy and information transfer rate (ITR) to functional text input capability.

**Figure 4:**
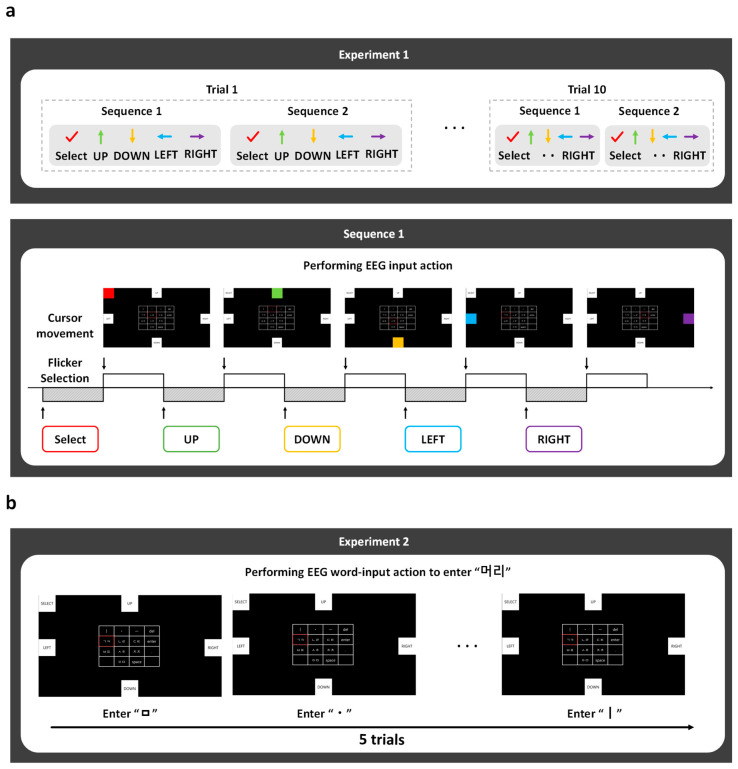
Experimental Procedure. (**a**) Experiment 1 designed to quantitatively evaluate the classification accuracy of five EEG-driven control commands (UP, DOWN, LEFT, RIGHT, and SELECT); (**b**) Experiment 2 designed to assess real-time Korean text entry performance through word input and sentence input tasks using EEG-based directional navigation within the Cheonjiin interface.

During both experiments, participants were seated approximately 70 cm from the display monitor and were instructed to perform an SSVEP-based speller task by visually attending to flickering stimuli to control cursor movements. Cursor navigation enabled the selection of consonants and vowels on the virtual Cheonjiin keyboard. Both experiments employed guided target selection tasks to ensure consistent task execution across participants. The experimental protocol and task flow are illustrated in Figure 4.

#### 2.4.1. Single-Step Command Recognition—Experiment 1

Experiment 1 was designed to evaluate the feasibility of the proposed EEG-based Cheonjiin speller by examining its ability to discriminate SSVEP-driven directional commands (UP, DOWN, LEFT, RIGHT, and SELECT) under controlled conditions. The primary objective was to validate the reliability of the core command decoding mechanism at the individual command level prior to implementing continuous text entry tasks.

Participants generated control inputs by fixating on visually presented flickering targets corresponding to each directional command. Each command was performed twice within a set, yielding a total of 10 selections per set. This procedure was repeated across 10 trials, as shown in Figure 4a. During each selection, classification outputs were continuously recorded to quantify recognition performance.

To mitigate visual fatigue and potential adaptation effects, a 2 s inter-command interval was introduced following each selection. Each participant completed 10 trials, and an additional rest period of approximately 30 s was provided between participants to maintain consistent experimental conditions.

#### 2.4.2. Multi-Step Korean Text Composition—Experiment 2

Experiment 2 aimed to investigate the usability of the proposed EEG-based Cheonjiin speller during continuous Korean text entry tasks. In contrast to the discrete command evaluation in Experiment 1, this experiment focused on real-time operation under sequential input conditions, assessing whether SSVEP-driven directional commands could be consistently translated into continuous cursor movement and character selection. In this context, text entry was performed through single, repeated, or sequential visual fixations following the compositional rules of the Cheonjiin keyboard.

Participants were instructed to input the Korean word “머리” (meaning “head”) by selecting the corresponding consonant and vowel elements using the SSVEP-controlled cursor on a virtual Cheonjiin interface. Character generation relied on both single and repeated selections, reflecting the inherent structure of the Cheonjiin layout. The word-entry task was repeated over five trials, with a 3 m rest interval between trials, as illustrated in Figure 4b.

During task execution, participants sequentially selected consonants and vowels and confirmed each syllable using the ‘enter’ key. Each command (UP, DOWN, LEFT, RIGHT, and SELECT) was treated as an individual classification decision. The predicted command was determined at every selection step, thereby enabling command-level evaluation of classification performance across the entire text entry process.

Based on the resulting classification outcomes, performance was quantified using accuracy, precision, recall, F1-score, and ITR, providing a comprehensive evaluation of both decoding performance and practical usability.

### 2.5. Data Analysis

To assess the performance of the proposed system, several standard evaluation metrics were employed based on confusion matrix analysis. The confusion matrix provides a structured representation of classification outcomes by comparing predicted labels with corresponding ground-truth labels. It consists of four basic components: true positive (TP), true negative (TN), false positive (FP), and false negative (FN). In this study, TP indicates correctly detected target commands, whereas FN refers to cases where intended commands were incorrectly classified. TN corresponds to correctly rejected non-target commands, and FP represents incorrect detection of non-target commands as target commands.

Using these definitions, multiple quantitative measures were calculated to evaluate classification performance. Accuracy was computed as the ratio of correctly classified samples to the total number of samples:

(5) $$Accuracy=\frac{TP+TN}{TP+TN+FP+FN}\;\times \;100$$

Precision measures the reliability of positive predictions:

(6) $$Precision=\frac{TP}{TP+FP}\;\times \;100$$

Recall reflects the system’s ability to correctly detect true target commands:

(7) $$Recall=\frac{TP}{TP+FN}\;\times \;100$$

Finally, the F1-score combines precision and recall into a single metric using their harmonic mean:

(8) $$F_{1}score=\frac{2\cdot Precision\cdot Recall}{Precision+Recall}$$

These metrics collectively provide insight into both classification accuracy and the consistency of command recognition in the proposed interface.

In addition to these classification metrics, the ITR was calculated to quantitatively evaluate the communication efficiency of the proposed system. The ITR, expressed in bits per minute (bits/min), was computed using the standard formula widely adopted in BCI and EMG-based communication studies:

(9) $$ITR={[log}_{2}N+P{log}_{2}P+(1-P){log}_{2}(\frac{1-P}{N-1})]\times \frac{60}{T}$$

where N is the number of available commands (five in our study), P denotes the classification accuracy, and T represents the average time required for one selection (in seconds). This metric jointly considers both recognition accuracy and selection speed, thereby providing an integrated measure of system efficiency for real-time operation.

## 3. Results

### 3.1. Spectral and Spatial Characteristics of SSVEP Responses

**Figure 5:**
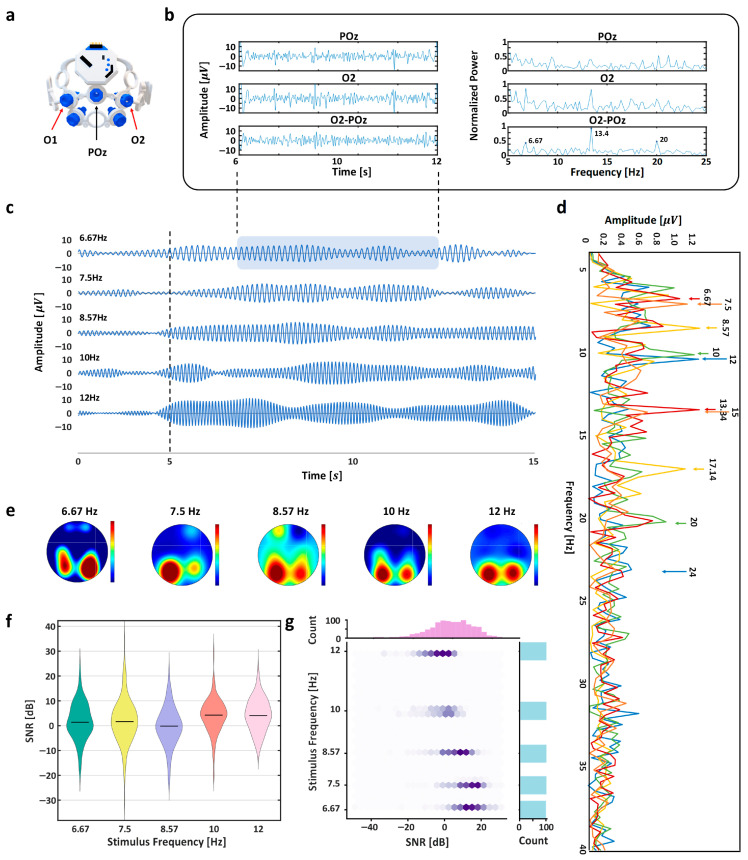
EEG signal analysis. (**a**) EEG headset and electrode configuration; (**b**) signal acquisition and processing procedure for 6.67 Hz stimulus; (**c**) time-domain EEG signals for each stimulus frequency (0~5 s: pre-stimulus; ≥5 s: stimulation); (**d**) Fast Fourier Transform (FFT) of EEG signals for each stimulus frequency; (**e**) scalp topographies showing frequency specific cortical activation; (**f**) signal-to-noise ratio (SNR) distributions for each stimulus frequency; (**g**) SNR values as a function of stimulus frequency.

As illustrated in Figure 5, the occipital EEG signals elicited by flicker stimulation were analyzed to verify frequency-specific cortical responses. The time-domain signals demonstrate relatively stable baseline activity during the pre-stimulus interval (0–5 s), followed by visually apparent oscillatory patterns after stimulus onset (≥5 s), reflecting entrainment to the presented flicker frequencies.

Frequency-domain analysis using Fast Fourier Transform (FFT) revealed distinct spectral peaks corresponding to each stimulation frequency. These peaks were consistently observed across conditions, indicating reliable frequency locking of cortical activity to the visual input. In addition to the fundamental frequency components, harmonic responses were also detectable, further supporting the presence of nonlinear neural synchronization mechanisms commonly associated with steady-state visual evoked potential (SSVEP).

Signal-to-noise ratio (SNR) evaluation further quantified the strength and distinctiveness of these frequency components. Additionally, to qualitatively illustrate the spatial distribution of frequency-specific responses, scalp topographical maps were generated using additional 6-channel EEG recordings acquired separately from the main experiment. These visualizations suggest that activation was predominantly localized over occipital regions, consistent with the cortical origin of visually evoked responses. The spatial distribution of frequency-specific power provides qualitative support for the physiological plausibility of the observed responses.

Together, the spectral amplitude, harmonic structure, SNR, and spatial distributions collectively confirm reliable frequency-specific SSVEP generation under the proposed stimulation paradigm, providing objective validation of the neural encoding mechanism underlying the directional control interface.

### 3.2. Event-Related Spectral Modulation of SSVEP Responses

To verify that the proposed system reliably captures frequency-specific neural responses, event-related spectral modulation induced by one of the stimulation frequencies (12 Hz) was examined as a representative example. The baseline interval was defined from −2 to 0 s, and intermittent stimulation was applied at 0–1 s, 2–3 s, 4–6 s, and 7–10 s.

**Figure 6:**
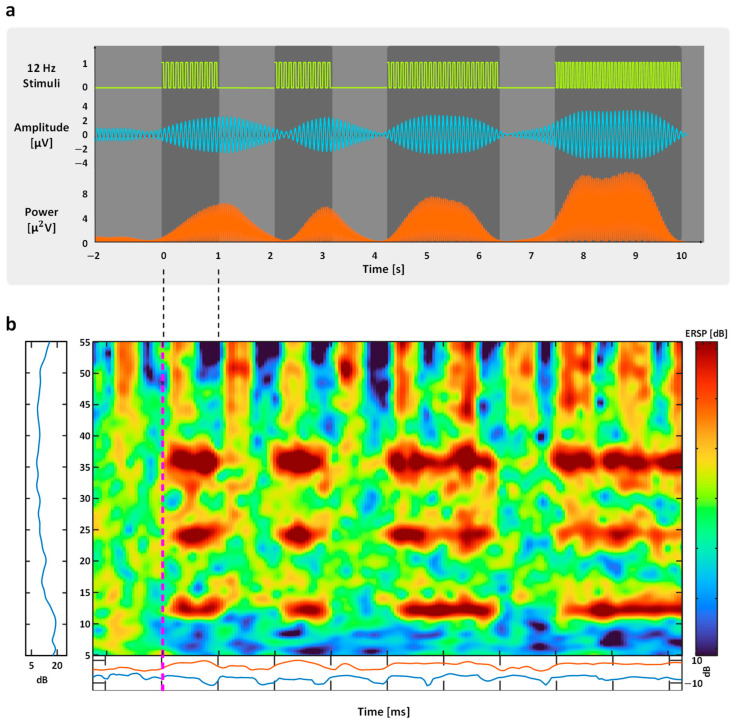
EEG signal analysis under 12 Hz flicker stimulation. (**a**) The temporal amplitude and corresponding spectral power changes induced by 12 Hz visual stimulation. The baseline was defined from −2 to 0 s, with stimulation applied intermittently at 0–1 s, 2–3 s, 4–6 s, and 7–10 s, showing stimulus-locked frequency enhancement relative to baseline. (**b**) Event-related spectral perturbation (ERSP) averaged across 10 trials under 12 Hz flicker stimulation.

As shown in Figure 6a, increases in signal amplitude and power were observed during each stimulation period, with responses time-locked to the visual flicker. These changes were transient and aligned with stimulus onset and offset, reflecting stimulus-driven modulation of neural activity.

The corresponding event-related spectral perturbation (ERSP) results (Figure 6b) showed consistent power enhancement at the stimulation frequency (12 Hz) across trials. The observed spectral changes were confined to the stimulation frequency and temporally aligned with the flicker presentation.

These results indicate that the proposed system can capture SSVEP responses at the target frequency.

### 3.3. Experimental Results

**Figure 7:**
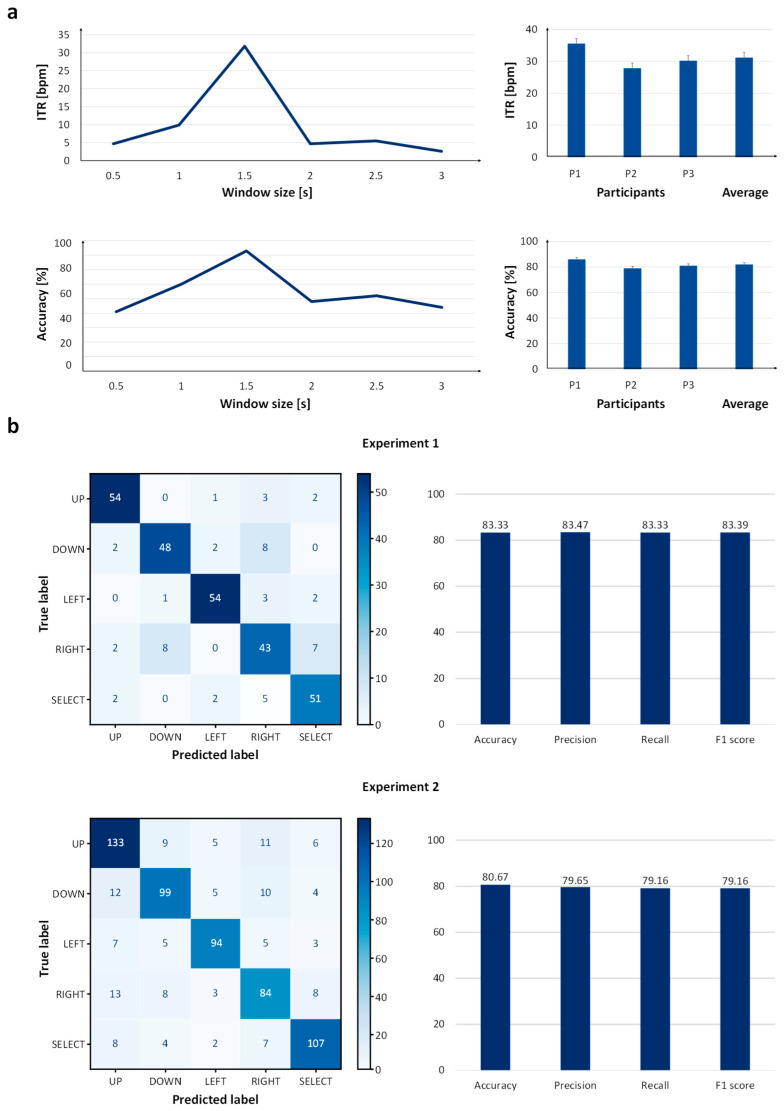
(**a**) Window size optimization results from the preliminary experiment, presenting accuracy and ITR values for different analysis window durations; (**b**) confusion matrices for Experiments 1 and 2 with corresponding bar charts of accuracy, precision, recall, and F1-score (%).

The preliminary experiment revealed a clear dependence of classification performance on the analysis window size. As shown in Figure 7a, both accuracy and information transfer rate (ITR) increased as the window size expanded from 0.5 s to 1.5 s. The highest performance was achieved at a window duration of 1.5 s, yielding an accuracy of 83% and an ITR of 31.8 bits/min.

However, further increasing the window size beyond 1.5 s resulted in a marked decline in performance. At window sizes of 2.0 s and above, accuracy decreased to below 52%, and ITR dropped substantially. These results indicate that a 1.5 s analysis window provides an optimal balance between sufficient temporal integration of SSVEP responses and minimal response latency. Accordingly, this window size was adopted for Experiments 1 and 2.

Two experimental protocols were designed to quantitatively assess the performance of the EEG-based Cheonjiin keyboard system. The first experiment evaluated single-command recognition, in which participants executed each of the five directional inputs—up, down, left, right, and select—independently to measure classification accuracy for individual actions. The second experiment examined sequential command execution, requiring participants to input multiple commands in succession to emulate realistic character entry scenarios and assess system stability during continuous operation. The classification performance of Experiments 1 and 2 was further examined in conjunction with the information transfer rate (ITR) to provide an integrated evaluation of system efficiency.

In Experiment 1, the confusion matrix presented in Figure 7b demonstrated strong diagonal dominance, indicating well-separated command recognition. The system achieved a mean accuracy of 83.33% ± 4.16% (95% CI: [73.0–93.7%]), with balanced precision (83.47%) and recall (83.33%), resulting in an F1-score of 83.39%. The observed classification performance was reflected in the corresponding ITR. In Experiment 1, the average ITR was 32.87 ± 5.26 bits/min (95% CI: [19.8–45.9] bits/min), indicating that higher classification accuracy was associated with increased communication throughput under repetitive command conditions.

In Experiment 2, classification performance was evaluated based on individual command predictions generated during the continuous text entry task. Although diagonal dominance remained evident, a moderate increase in off-diagonal elements was observed, indicating slightly higher misclassification during continuous text entry tasks. This resulted in a mean accuracy of 80.67% ± 2.75% (95% CI: [73.8–87.5%]), precision of 79.65%, recall of 79.16%, and F1-score of 79.16%. Classification performance during sustained operation was accompanied by a corresponding adjustment in ITR, highlighting the sensitivity of communication speed to accuracy under continuous spelling conditions. The mean ITR in Experiment 2 was 29.53 ± 2.70 bits/min (95% CI: [22.8–36.2] bits/min), reflecting the influence of task complexity on overall communication efficiency.

When averaged across both experiments, the system achieved a mean accuracy of 82.00%, a mean precision of 81.56%, a mean recall of 81.25%, and a mean F1-score of 81.28%. Importantly, the relatively small differences between precision and recall indicate that the classifier maintained balanced performance without strong bias toward specific commands. Also, the overall average ITR was 31.2 bits/min.

A Wilcoxon signed-rank test was conducted to compare the performance between Experiment 1 and Experiment 2. Although all subjects showed higher accuracy and ITR values in Experiment 1, the differences were not statistically significant (accuracy: *p* = 0.25, ITR: *p* = 0.25).

Because the ITR depends not only on accuracy but also on the number of available commands and the selection time per decision, these results indicate that the proposed five-command SSVEP paradigm can support a moderate communication rate under the tested conditions. Under continuous spelling conditions, command-level discrimination remained observable, suggesting potential applicability to real-time Korean text entry.

These findings suggest that the proposed framework maintains consistent recognition performance even in the presence of inter-subject differences in signal amplitude, duration, and activation patterns. Importantly, classification was performed using only CCA without the use of more advanced methods.

### 3.4. Longitudinal Experiment

To evaluate session-to-session stability, additional experiments were conducted across four consecutive days without recalibration. The same experimental protocol used in Experiments 1 and 2 was repeated with each participant under consistent conditions.

**Figure 8:**
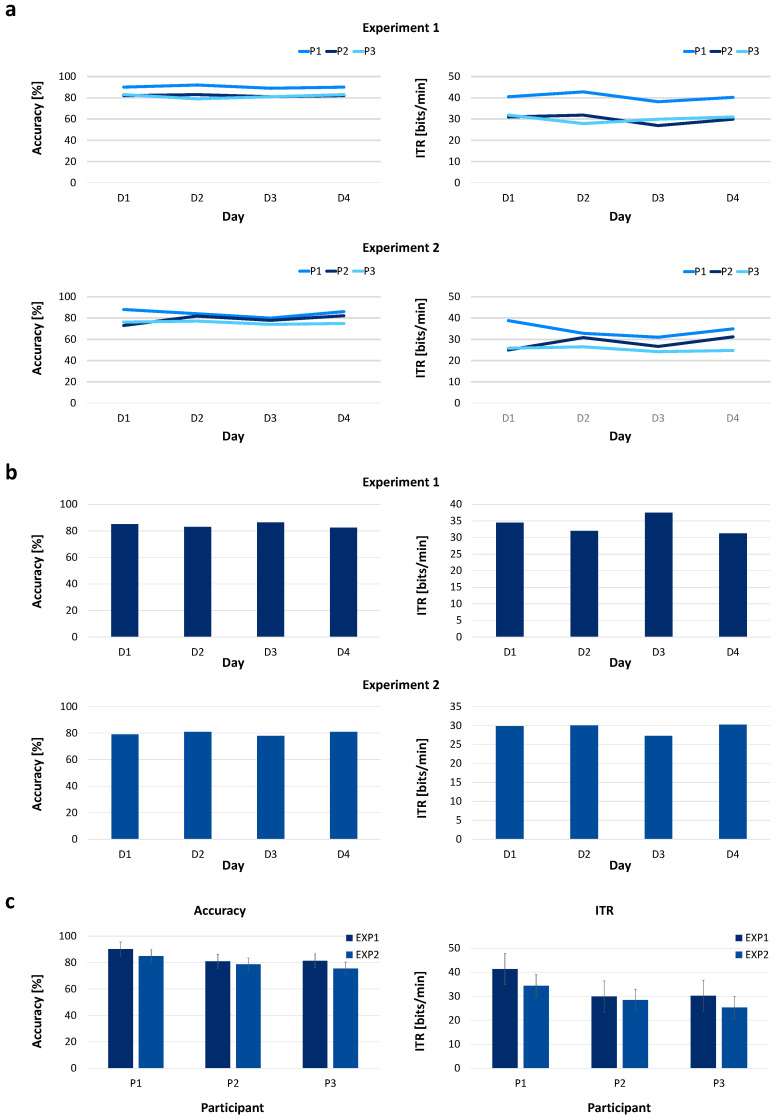
Longitudinal evaluation of system performance across multiple sessions. (**a**) Longitudinal changes across four consecutive days (D1–D4) for each participant in Experiment 1 and Experiment 2; (**b**) day-wise group averages of performance across participants; (**c**) experiment-wise comparison showing mean accuracy and information transfer rate (ITR) across participants for Experiment 1 and Experiment 2.

Figure 8 presents the classification accuracy (%) and information transfer rate (ITR, bits/min) measured across sessions. In Experiment 1, the accuracy remained relatively consistent across four consecutive days, with values of 85%, 83%, 86.33%, and 82.32% from Day 1 to Day 4, respectively. The corresponding ITR values were 34.43, 31.99, 37.51, and 31.22 bits/min. Averaged across sessions, the mean accuracy was 84.17% ± 5.30% (95% CI: [71.0%, 97.3%]), and the mean ITR was 33.79 ± 6.48 bits/min (95% CI: [17.7, 49.9] bits/min). Subject-wise results showed consistent patterns, with individual accuracy generally ranging between 80% and 85%.

In Experiment 2, accuracy also remained relatively consistent across days, with values of 79.05%, 80.98%, 77.94%, and 80.97% from Day 1 to Day 4. The corresponding ITR values were 29.86, 30.04, 27.28, and 30.26 bits/min. The mean accuracy across sessions was 79.74% ± 4.85% (95% CI: [67.7%, 91.7%]), while the mean ITR was 29.36 ± 4.57 bits/min (95% CI: [18.0, 40.7] bits/min). Subject-wise analysis indicated moderate inter-subject variability, although overall trends were similar across sessions.

These findings suggest that the proposed system can operate across multiple sessions without recalibration, although further evaluation with a larger sample size is required to confirm long-term stability.

### 3.5. User Workload Assessment

**Figure 9:**
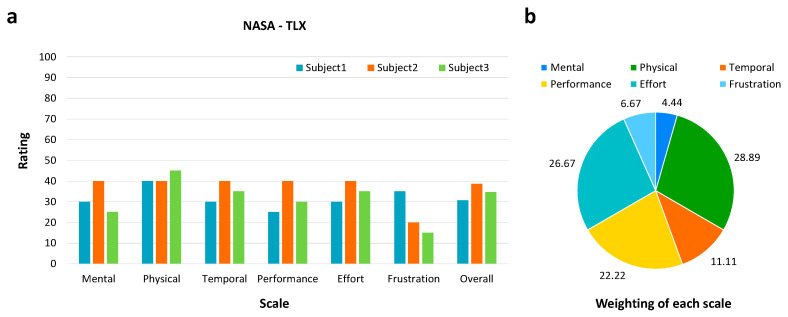
NASA-TLX for user workload assessment. (**a**) Bar chart comparing NASA-TLX scores across subjects for Experiments 1 and 2; (**b**) pie chart representing relative contribution of each NASA-TLX subscale to total workload.

To further assess user workload during system operation, a subjective evaluation was conducted using the NASA Task Load Index (NASA-TLX), which includes six subscales: mental demand, physical demand, temporal demand, performance, effort, and frustration. As shown in Figure 9, the overall workload score was 34.67 ± 4.00, indicating a relatively low level of perceived workload during system operation.

The average subscale scores were as follows: mental demand: 31.67; physical demand: 41.67; temporal demand: 35; performance: 31.67; effort: 35; and frustration: 23.33. The weighted analysis indicated that physical demand and effort contributed most to the overall workload, followed by performance, while mental demand and frustration had relatively lower contributions.

## 4. Discussion

This study developed an EEG-based Cheonjiin speller system employing a four-directional input structure (up, down, left, and right). Its input recognition performance and practical usability were quantitatively assessed through experiments involving human participants. The system achieved a mean accuracy of 83.33% ± 4.16% and 80.67% ± 2.75% in Experiment 1 and 2. The corresponding ITR values were 32.87 ± 5.26 bits/min and 29.53 ± 2.70 bits/min.

Although Experiment 1 yielded higher accuracy and ITR values than Experiment 2 for all subjects, statistical analysis using the Wilcoxon signed-rank test indicated that the differences were not statistically significant (*p* = 0.25). Experiment 1 involved discrete single-command recognition, whereas Experiment 2 required continuous sequential input for text entry. The increased task complexity in Experiment 2 may have introduced additional cognitive load and required sustained attention over longer periods, which could affect the stability of SSVEP responses.

In addition, continuous operation may increase susceptibility to temporal fluctuations and error accumulation, as incorrect selections can influence subsequent inputs. These factors may contribute to the relatively lower accuracy and ITR observed in Experiment 2 compared to Experiment 1.

The average ITR of 31.2 bits/min is lower than that reported in several recent SSVEP-based speller systems, where ITR values often exceed 60–100 bits/min [50]. However, such systems typically rely on a large number of targets, multi-channel EEG configurations, and advanced decoding algorithms to maximize communication speed. In contrast, SSVEP-based spellers designed with a limited number of targets (typically 4–8 commands), reduced EEG channels (e.g., 1–3 channels), and conventional CCA-based decoding methods generally report ITR values in the range of approximately 20–50 bits/min [51,52]. In addition, stepwise or multi-stage input strategies have been employed in such systems to reduce command complexity while maintaining usability, although this often introduces constraints on input speed.

These observations highlight a trade-off between system simplicity and communication speed, where a reduced number of commands simplifies the system design but limits input speed, whereas increasing the number of commands may increase system complexity [18,29,50]. In this context, the performance of the proposed system falls within the expected range for low-complexity SSVEP-based spellers. The use of five commands and a two-channel EEG configuration aligns with prior studies that prioritize reduced hardware requirements and simplified system design. The 1.5 s analysis window introduces inherent latency, while the limited number of commands constrains the maximum achievable information transfer rate.

Whereas many previous studies have relied on deep learning-based architectures for complex EEG signal modeling, the present study demonstrates that reliable discrimination of directional and selection commands can be accomplished using only CCA, without reliance on computationally intensive deep learning models [53,54]. The resulting lightweight framework supports real-time operation and remains feasible for implementation on low-performance hardware platforms.

Despite these advantages, the current implementation based on CCA has several limitations. More advanced methods such as filter bank canonical correlation analysis (FBCCA) may improve signal-to-noise ratio and enable shorter analysis windows, potentially enhancing both accuracy and ITR [55,56]. In addition, the current signal processing pipeline does not explicitly address artifacts caused by involuntary muscle movements, which may affect system performance in practical use scenarios. Incorporating artifact mitigation techniques will be necessary for more robust operation, particularly in real-world environments.

In addition, the proposed system provides a promising communication approach for individuals with limited limb mobility and may be extended to clinical applications with further validation. By integrating the Cheonjiin keyboard layout—characterized by a reduced key set and shorter transition distances compared to QWERTY—Korean text entry can be performed using directional control commands alone. The reduction in required input transitions is expected to lower user burden. Furthermore, electrode placement and command mapping were determined based on anatomical considerations, contributing to consistent performance despite inter-subject variability in signal amplitudes and activation patterns.

However, this result may be related to the limited sample size (*n* = 3), which restricts statistical power. The observed performance difference is likely associated with increased task complexity in continuous text entry conditions rather than random variation. Accordingly, the present study should be interpreted as a proof-of-concept, and further validation with a larger participant population is required to improve statistical reliability and generalizability.

The system operates using two occipital EEG channels (O1 and O2), which reduces hardware complexity compared to multi-channel configurations while still capturing responses from bilateral visual cortical regions. Further investigation into single-channel configurations is warranted to evaluate whether comparable performance can be achieved with reduced system complexity. It may reflect a trade-off between system simplicity and signal robustness, as a single-channel configuration could further reduce hardware complexity, it may potentially affect classification stability.

From a usability perspective, factors such as user fatigue, calibration requirements, and real-time deployment constraints should also be considered. In addition, calibration procedures required prior to system use may introduce additional setup time and variability across users, which could affect practical usability [57,58]. The NASA-TLX results indicate that physical demand and effort were the primary contributors to the overall workload, while mental demand and frustration showed relatively lower contributions. This pattern suggests that the task required continuous operational engagement rather than imposing substantial cognitive or emotional strain. Such a tendency may be related to the characteristics of SSVEP-based interaction, which relies on sustained visual attention and repetitive gaze fixation. These findings highlight the importance of optimizing visual stimulation parameters and interaction strategies to reduce user fatigue and improve long-term usability. Real-time deployment may also be influenced by processing latency and system responsiveness, particularly under continuous operation conditions.

The use of relatively low stimulation frequencies (6.67–12 Hz) in this study may contribute to visual fatigue and discomfort during prolonged use and may also pose potential safety concerns for photosensitive individuals. These frequencies were selected based on practical design considerations, including compatibility with a 60 Hz display refresh rate, harmonic relationships between stimulation signals, and the goal of maintaining a low-cost and accessible system configuration. In future work, higher-frequency stimulation or alternative paradigms, such as code-modulated SSVEP, will be investigated to reduce visual fatigue while maintaining reliable classification performance.

Overall, this study underscores the feasibility of EEG-based brain–computer interaction (BCI) systems as accessible communication tools. The findings provide a foundation for future user-centered interface development aimed at improving communication autonomy and information accessibility for individuals with physical limitations.

## 5. Study Limitations

This study has several limitations. The current study was designed as a proof-of-concept to evaluate the feasibility of the proposed system under controlled conditions, and therefore, a limited number of participants were included. The sample size was relatively small (*n* = 3), which limits generalizability and statistical power, although consistent trends were observed across participants; future studies will include a larger cohort. The system employs a limited number of commands, which constrains input speed, and increasing the number of commands or adopting alternative input strategies may improve efficiency at the cost of increased complexity. In addition, potential artifacts caused by involuntary muscle activity were not explicitly addressed and may be mitigated through advanced preprocessing or adaptive filtering techniques. Finally, the system has not been evaluated in clinical populations, and future work will involve experiments with users with motor impairments to assess real-world applicability.

## 6. Conclusions

In this study, a Cheonjiin-based SSVEP speller using a two-channel EEG configuration was developed for Korean text input. The system achieved an average accuracy of 82.00% and an average ITR of 31.2 bits/min, with relatively consistent performance across subjects.

Although the performance is lower than that reported in some recent SSVEP-based speller systems, the proposed approach reflects a trade-off between communication speed and system simplicity. The reduced number of EEG channels and the structured input method may be advantageous in applications where ease of setup and operational simplicity are important.

The achieved performance, while moderate, suggests that the proposed system may be suitable for assistive communication scenarios where system simplicity and ease of setup are important considerations. In particular, the use of a minimal number of EEG channels and a structured input method may facilitate practical deployment in environments with limited resources or user tolerance. While the achieved ITR is lower than that of high-performance SSVEP spellers, it falls within the range reported for simple and low-channel BCI systems, suggesting that the proposed approach may be suitable for applications prioritizing usability over maximum communication speed.

Future work will focus on improving classification performance through advanced signal processing methods, exploring configurations with a greater number of commands, and conducting validation with larger and more diverse participant groups, including clinical populations.

## Data Availability

The original contributions presented in the study are included in the article, further inquiries can be directed to the corresponding authors.
